# Prognostic and Diagnostic Value of Node-RADS for Non-Small Cell Lung Cancer Following Neoadjuvant Therapy: A Multicenter Cohort Study

**DOI:** 10.3390/diagnostics16132021

**Published:** 2026-06-29

**Authors:** Jianyu Wang, Yuhang Wang, Hao Chen, Han Zhang, Xiaojiang Zhao, Qiuqiao Mu, Yuhang Jiang, Yanbo Wang, Zhenchun Song, Yixing Li, Xin Li, Daqiang Sun

**Affiliations:** 1 Graduate School, Tianjin Medical University, Tianjin 300070, China; wangjianyu0536@163.com (J.W.);; 2 Thoracic Surgery Clinical College, Tianjin Medical University, Tianjin 300070, China; 3 Department of Thoracic Surgery, Tianjin Chest Hospital, Tianjin 300222, China; wangyh_up@foxmail.com (Y.W.);; 4 Department of Thoracic Surgery, Beijing Chest Hospital, Beijing 101149, China; 5 Graduate School, Tianjin University, Tianjin 300072, China; 6 Department of Imaging, Tianjin Chest Hospital, Tianjin 300222, China; 7 Department of Thoracic Surgery, The First Affiliated Hospital of Xi’an Jiaotong University, Xi’an 710061, China

**Keywords:** non-small cell lung cancer, Node-RADS, neoadjuvant therapy, lymph nodes, computed tomography

## Abstract

**Background/Objectives:** The aim of this study was to evaluate the performance of the Node-RADS scoring system for predicting lymph node metastasis in patients with non-small cell lung cancer (NSCLC) after neoadjuvant therapy and to assess its prognostic value for overall survival (OS) and event-free survival (EFS). **Methods:** A total of 247 patients with non-small cell lung cancer (NSCLC) from three centers who underwent surgery after neoadjuvant therapy were retrospectively enrolled. Post-treatment Node-RADS scores were reassessed by radiologists based on preoperative contrast-enhanced CT images. Logistic regression analysis was used to evaluate the predictive value of Node-RADS for postoperative pathological lymph node metastasis, while Cox regression analysis was performed to assess its associations with OS and EFS. Kaplan–Meier analysis was used to compare survival differences among different Node-RADS risk groups. **Results:** A total of 247 patients were included in this study, comprising 211 men and 36 women, with a mean age of 63.40 ± 7.58 years. Post-treatment Node-RADS score was significantly associated with both OS and EFS. In multivariable Cox regression analysis, Node-RADS remained independently associated with OS (HR = 1.79, 95% CI: 1.50–2.15, *p* < 0.001) and EFS (HR = 1.41, 95% CI: 1.23–1.62, *p* < 0.001). Using a Node-RADS score of 3 as the cutoff value, patients in the high-risk group had significantly worse OS and EFS than those in the low-risk group (both *p* < 0.01). For the prediction of lymph node metastasis, the inclusion of post-treatment Node-RADS markedly improved the discriminatory performance of the model, with an AUC of 0.769, a sensitivity of 46.2%, and a specificity of 87.6%. **Conclusions:** The Node-RADS score may provide useful imaging information for patient-level assessment of residual lymph node metastasis risk and survival stratification in patients with NSCLC after neoadjuvant therapy. These findings suggest that the scoring system may support patient-level post-treatment risk assessment.

## 1. Introduction

Lung cancer remains the leading cause of cancer-related death worldwide [1]. In lung cancer staging systems, lymph node involvement plays a decisive role in prognosis and treatment decision-making [2]. In recent years, the use of neoadjuvant therapy in resectable locally advanced NSCLC has gradually increased, providing some patients with greater opportunities for curative resection and improved long-term outcomes [3]. However, in clinical practice, responses to neoadjuvant therapy vary considerably among patients. In particular, assessment of post-treatment lymph node status and residual tumor burden remains a key issue affecting subsequent surgical planning, pathological evaluation, and prognostic stratification [4]. Therefore, accurate evaluation of regional lymph node involvement is of great importance. Computed tomography (CT) is the most commonly used imaging modality for regional lymph node staging in NSCLC [5]. However, lymph node staging becomes more challenging in the setting of neoadjuvant therapy. Malignant lymph nodes are not necessarily enlarged, whereas benign lymph nodes may show reactive enlargement due to post-treatment inflammatory changes, necrotic repair, and fibrosis, resulting in substantial overlap in imaging findings. At the same time, conventional size-based criteria for lymph node assessment lack a unified threshold, which further compromises the consistency and accuracy of staging evaluation [5,6]. Therefore, current imaging-based assessment of lymph node status after neoadjuvant therapy still has clear limitations, and a standardized, reproducible radiological method with high diagnostic performance is still lacking in clinical practice.

In the field of radiologic assessment, Reporting and Data Systems (RADS) have been widely used to promote the standardization and consistency of imaging reports. On this basis, the Node Reporting and Data System (Node-RADS) was proposed to stratify the risk of lymph node involvement based on CT or magnetic resonance imaging (MRI) findings [7]. Node-RADS uses a 5-point scoring system, with each category representing an increasing probability of malignancy. The scoring is mainly based on a comprehensive assessment of lymph node size and morphologic features. In recent years, several studies have begun to explore the value of Node-RADS in lymph node assessment for NSCLC, although the reported diagnostic performance has been somewhat inconsistent across studies [8,9]. Unlike more widely established RADS, Node-RADS remains an evolving imaging scoring system, and further validation is still needed before it can be considered a universally reliable tool for lymph node assessment.

In patients with NSCLC, a variety of clinical, pathological, and treatment-related factors may affect postoperative survival outcomes. Among these, lymph node status has consistently been regarded as an important determinant of prognosis and recurrence risk [4,10]. Recently, a limited number of studies have focused on the application of Node-RADS and related imaging models in the assessment of lymph nodes in lung cancer, suggesting that CT-based structured or quantitative assessment methods may not only improve the imaging diagnosis of lymph node metastasis, but also provide additional information for subsequent risk stratification [9]. Overall, however, evidence regarding the diagnostic and prognostic value of Node-RADS in NSCLC after neoadjuvant therapy remains limited, particularly in the absence of systematic evaluation based on multicenter cohorts. Therefore, in this multicenter cohort, we aimed to analyze the predictive performance of post-treatment Node-RADS scores for lymph node metastasis and further investigate their associations with OS and EFS. In addition, postoperative pathological findings were used as the reference standard to evaluate diagnostic performance, with the goal of providing more reliable imaging-based evidence for preoperative risk stratification and clinical decision-making.

## 2. Materials and Methods

### 2.1. Study Design and Patients

**Figure 1:**
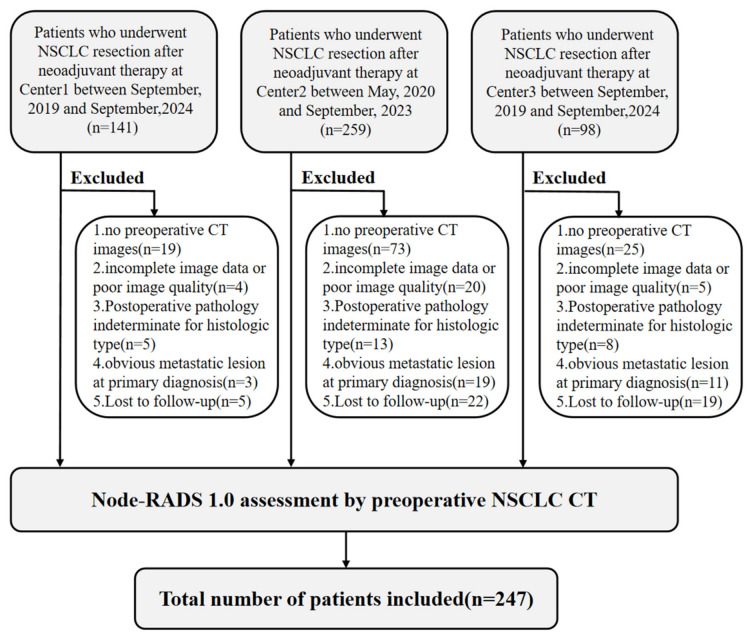
Flowchart of patient inclusion and exclusion.

This study was approved by the ethics committee, and the requirement for informed consent was waived owing to its retrospective design. We retrospectively reviewed the data of patients treated at Center 1, Center 2, and Center 3 between September 2019 and September 2024. Patients were eligible regardless of their baseline clinical nodal status before neoadjuvant therapy; therefore, both clinically node-negative and clinically node-positive patients were included in this study. Patients who met the following inclusion criteria were enrolled: (1) receipt of at least one cycle of neoadjuvant therapy followed by radical pulmonary resection and systematic lymph node dissection; and (2) postoperative pathological confirmation of NSCLC. The exclusion criteria were as follows: (1) absence of preoperative CT imaging data; (2) poor-quality CT images; (3) inability to determine the histological type based on postoperative pathological examination; (4) distant metastasis (M1); and (5) missing postoperative follow-up information. Ultimately, 247 eligible patients were included in the study. Figure 1 shows the flowchart of the inclusion and exclusion criteria used in this study.

### 2.2. Neoadjuvant Therapy

All enrolled patients received preoperative neoadjuvant therapy after evaluation by senior oncology specialists. Treatment decisions were individualized in accordance with the Chinese Medical Association Guidelines for Clinical Diagnosis and Treatment of Lung Cancer (2024 Edition) [11] and the NCCN recommendations for perioperative systemic therapy in non-small cell lung cancer [12], while also taking into account clinical stage, histological subtype, performance status, organ function, and drug accessibility. Neoadjuvant treatment consisted of either neoadjuvant chemotherapy alone or neoadjuvant chemoimmunotherapy. All chemotherapy regimens were platinum-based doublets, mainly including paclitaxel plus platinum, pemetrexed plus platinum, and gemcitabine plus platinum. For patients receiving combined immunotherapy, a PD-1 inhibitor was added to the platinum-based doublet chemotherapy regimen, mainly including pembrolizumab, tislelizumab, serplulimab, camrelizumab, and sintilimab. After completion of the planned neoadjuvant therapy, patients who were reassessed as suitable for surgery and had no clear surgical contraindications subsequently underwent surgical treatment. The specific surgical approach was individualized according to each patient’s tumor characteristics, post-treatment evaluation findings, and overall condition, and included either thoracoscopic surgery or open thoracotomy. The final surgical procedure was determined after thorough discussion by the thoracic surgery team to ensure surgical safety while minimizing patient trauma as much as possible.

### 2.3. Data Collection

Clinical data were extracted from the electronic medical record system, including sex, age, history of hypertension, history of diabetes mellitus, history of coronary heart disease, history of cerebral infarction, clinical stage, pathological type, surgical approach, intraoperative blood loss, neoadjuvant treatment regimen, chemotherapy regimen, number of neoadjuvant treatment cycles, neoadjuvant treatment-related complications, postoperative pathological lymph node metastasis status, OS and EFS.

### 2.4. CT Examination and Node-RADS Assessment

All patients underwent contrast-enhanced chest CT after neoadjuvant therapy and before surgery. Imaging data were acquired using multidetector CT scanners from the three participating centers, including Philips & Neusoft Medical Systems (PNMS), Siemens, and Philips systems. As this was a multicenter retrospective study, specific scanner models and acquisition protocols varied somewhat across centers. However, all examinations were performed using routine clinical contrast-enhanced chest CT protocols and met the requirements for Node-RADS assessment of lymph node size and morphologic features. The technical parameters included a tube voltage of 120 kVp, automatic tube current modulation, a slice thickness of approximately 1.0 mm, a rotation time of 0.4–0.5 s, a pitch of approximately 0.6–1.4, and an image reconstruction matrix of 512 × 512. Contrast-enhanced scanning was performed after intravenous injection of a nonionic iodinated contrast agent, with saline flushing administered before and after injection when necessary. Thin-section enhanced images were reconstructed in multiple planes, including axial, coronal, and sagittal images, for subsequent lymph node assessment.

**Figure 2:**
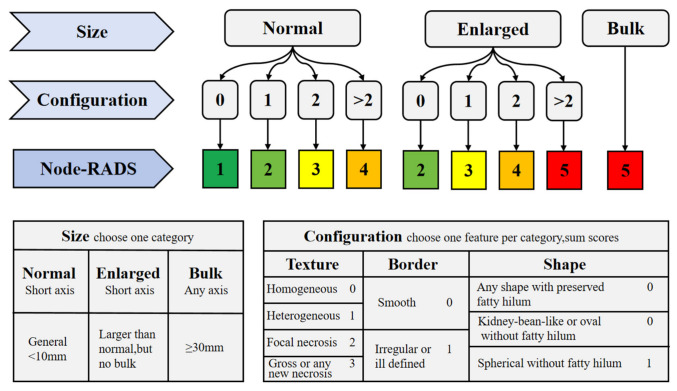
Node-RADS assessment flowchart for regional lymph nodes in non-small cell lung cancer.

To ensure the consistency and accuracy of lymph node evaluation in this study, two radiologists with 6 and 11 years of experience in thoracic imaging, respectively, received structured training on the Node-RADS scoring system before image review. They then independently reviewed all chest CT images while blinded to the pathological results and assigned Node-RADS scores according to lymph node size and morphology (Figure 2). Node-RADS assessment was performed on a per-patient basis; for each patient, the regional lymph node with the highest suspicion on post-treatment CT was used to determine the final patient-level Node-RADS score. Specifically, contrast-enhanced chest CT was selected for Node-RADS assessment because it provides optimal visualization of lymph nodes.

**Figure 3:**
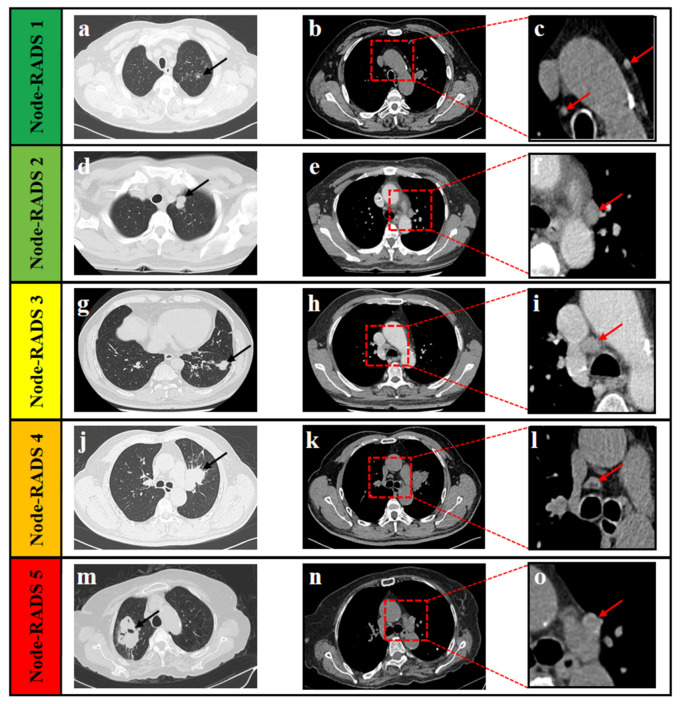
Representative CT images of Node-RADS categories 1–5. Black arrows indicate primary tumors; red boxes indicate selected regional lymph nodes; red arrows indicate magnified lymph-node views. Images (**a**–**c**), (**d**–**f**), (**g**–**i**), (**j**–**l**), and (**m**–**o**) correspond to Node-RADS categories 1, 2, 3, 4, and 5, respectively.

Node-RADS 1.0 was used for evaluation, with scores assigned to reflect the likelihood of lymph node metastasis: 1 (very low), 2 (low), 3 (equivocal), 4 (high), and 5 (very high) (see Figure 3 for details). Briefly, the Node-RADS score is based on two main assessment criteria: Size and Configuration. Under the Size criterion, lymph nodes with a short-axis diameter ≥10 mm were considered “enlarged”, and lymph nodes with any axis ≥30 mm were considered “bulk”. This constituted the first step of the assessment, in which lymph nodes were categorized as “normal” “enlarged” or “bulk”. The Configuration criterion included three subcategories: texture: homogeneous (0 points), heterogeneous (1 point), focal necrosis (2 points), and gross necrosis (3 points); border: smooth (0 points) or irregular/ill-defined (1 point); and shape: any shape with a preserved fatty hilum (0 points), reniform or oval shape without a fatty hilum (0 points), and spherical shape without a fatty hilum (1 point). In the second step, the total Configuration score was calculated as the sum of the highest score in each subcategory, ranging from 0 to 5 points. Finally, this total score was weighted according to Size and Configuration to determine the final Node-RADS score. If disagreement arose between the two readers regarding the final Node-RADS score, a consensus was reached through discussion. If disagreement persisted, a third radiologist with extensive experience in thoracic imaging made the final decision.

### 2.5. Follow-Up

All patients were followed up regularly after the initiation of treatment. According to the follow-up protocol, patients were mainly monitored through outpatient visits or telephone contact. Follow-up CT scans were performed every 3 months during the first 2 years and every 6–12 months during years 3–6. Follow-up duration was calculated from the initiation of neoadjuvant therapy to the date of last follow-up or death. The primary survival endpoint was OS defined as the time from the initiation of neoadjuvant therapy to death from any cause or the last follow-up. The secondary endpoint was EFS defined as the time from the initiation of neoadjuvant therapy to the first occurrence of disease progression, recurrence, metastasis, or death.

### 2.6. Statistical Analysis

Data were analyzed using SPSS version 25 (IBM Corp., Armonk, NY, USA). According to data distribution, continuous variables were expressed as mean ± standard deviation, whereas categorical variables were presented as counts and percentages. For between-group comparisons, Student’s *t* test was used for normally distributed data, the Mann–Whitney U test was used for non-normally distributed data, and the chi-square test or Fisher’s exact test was used for categorical variables. Interobserver agreement between the two radiologists for the initial Node-RADS scores before consensus was assessed using quadratic weighted Cohen’s kappa.

ROC curve analysis was performed to evaluate the discriminatory ability of the diagnostic model, and ROC analyses for OS and EFS were performed based on survival/event status at the last follow-up. The Node-RADS score was entered into the Cox regression model as a continuous variable, whereas in Kaplan–Meier survival analysis it was categorized according to a prespecified cutoff value. Considering that a Node-RADS score of 3 indicates suspicious lymph node status, whereas scores of 4–5 suggest a high risk of malignancy, patients with a Node-RADS score ≥ 3 were defined as the high-risk group and those with a score < 3 as the low-risk group. Survival differences between groups were compared using the log-rank test. Univariable Cox proportional hazards regression analysis was used to assess the associations of clinical variables and post-treatment Node-RADS score with OS and EFS. Subsequently, based on the univariable analysis results, study objectives, and clinical judgment, candidate variables were entered into multivariable Cox regression models to identify independent prognostic factors. Variables with *p* < 0.05 in univariable logistic regression analyses and variables with *p* < 0.10 in univariable Cox regression analyses were considered for multivariable models. Clinically relevant variables, such as age, were retained for adjustment when appropriate. To reduce potential overfitting, a parsimonious modeling strategy was adopted according to clinical relevance and the number of outcome events. The results were reported as hazard ratios (HRs) with 95% confidence intervals (95% CIs).

For the diagnostic analysis, postoperative pathological lymph node metastasis was defined as a binary patient-level endpoint. Univariable logistic regression analysis was used to evaluate the associations between each variable and postoperative pathological lymph node metastasis. For descriptive category-specific analyses, Node-RADS category 1 was used as the reference, and odds ratios for individual Node-RADS categories were estimated using a 0.5 continuity correction because some categories contained no pN− cases. Candidate variables were then selected on the basis of the univariable analysis results, clinical relevance, and the principle of model parsimony, and were entered into the multivariable logistic regression model. Because clinical stage already includes lymph node staging information, whereas post-treatment Node-RADS mainly reflects lymph node involvement status, clinical stage was not included as a candidate variable in either the logistic or Cox models to avoid potential information overlap that might interfere with the assessment of the independent predictive value of Node-RADS. On this basis, a clinical model and a clinical plus post-treatment Node-RADS model were constructed separately, and nomograms were developed to visually illustrate the contribution of each variable to the prediction of outcome events.

## 3. Results

### 3.1. Clinical Characteristics of the Patients

A total of 247 patients with NSCLC who underwent surgery after neoadjuvant therapy were included in this study, with a mean age of 63.40 ± 7.58 years. Squamous cell carcinoma was the predominant histological subtype, accounting for 192 cases (77.7%) whereas adenocarcinoma was identified in 55 cases (22.3%). According to the Tumor-Node-Metastasis staging system, 25 patients (10.1%) were classified as stage IB, 23 (9.3%) as stage IIA, 40 (16.2%) as stage IIB, 103 (41.7%) as stage IIIA, and 56 (22.7%) as stage IIIB. All patients received neoadjuvant therapy, including 18 patients (7.3%) who received neoadjuvant chemotherapy alone and 229 patients (92.7%) who received neoadjuvant chemoimmunotherapy. The most commonly used chemotherapy regimen was paclitaxel plus platinum, which was administered in 200 patients (81.0%) followed by pemetrexed plus platinum in 42 patients (17.0%) and gemcitabine plus platinum in 5 patients (2.0%). The baseline characteristics of the patients are presented in Table 1. A total of 3321 lymph nodes were pathologically examined, with a median of 13 per patient (IQR, 9–18). The interobserver agreement for the initial Node-RADS scores was good, with a quadratic weighted Cohen’s κ value of 0.837 (95% CI: 0.781–0.879).

### 3.2. Predictive Value of Post-Treatment Node-RADS Combined with Clinical Baseline Variables for Lymph Node Metastasis

Univariable logistic regression analysis showed that pathological type (*p* = 0.013), number of neoadjuvant treatment cycles (*p* = 0.029), chemotherapy regimen (*p* = 0.014), complications of neoadjuvant therapy (*p* = 0.040), and post-treatment Node-RADS (*p* < 0.001) were associated with lymph node metastasis and were therefore included as candidate variables (Supplementary Table S1). Further multivariable logistic regression analysis revealed that only post-treatment Node-RADS remained significantly associated with lymph node metastasis (*p* < 0.001) suggesting its potential value for patient-level assessment of lymph node status after neoadjuvant therapy. A descriptive patient-level analysis showed that the frequency of pN+ disease increased across Node-RADS categories, from 0.8% in category 1 and 28.8% in category 2 to 100.0% in categories 3–5 (Supplementary Table S2). Category-specific ORs are also provided in Supplementary Table S2; because Node-RADS categories 3–5 contained no pN− cases, these estimates should be interpreted descriptively. Considering the clinical relevance of age, the final prediction model included both age and post-treatment Node-RADS, and a nomogram was constructed accordingly (Supplementary Figure S1). The results of the univariable and multivariable analyses are shown in Supplementary Table S3.

**Figure 4:**
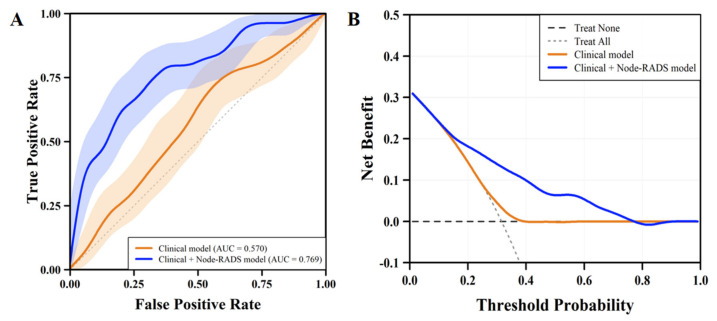
Diagnostic performance of Node-RADS for predicting lymph node metastasis. (**A**) Receiver operating characteristic curves of models with and without Node-RADS. (**B**) Decision curve analysis of models with and without Node-RADS.

To further evaluate the incremental predictive value of post-treatment Node-RADS, we compared the performance of the clinical model with that of the clinical plus post-treatment Node-RADS model. The results showed that after incorporating post-treatment Node-RADS, the AUC increased to 0.769, with a marked improvement in sensitivity compared with the clinical model, although the absolute sensitivity remained moderate. In contrast, although the clinical model without post-treatment Node-RADS showed relatively high specificity, its ability to identify positive cases was poor, as reflected by its very low sensitivity. As shown in Table 2, the receiver operating characteristic (ROC) curves and decision curve analysis (DCA) curves are presented in Figure 4. These findings suggest that clinical baseline information alone is insufficient to effectively identify patients with residual lymph node metastasis after neoadjuvant therapy, whereas the incorporation of Node-RADS improved the discriminatory performance of the model.

### 3.3. Cox Regression Analysis of Post-Treatment Node-RADS in Relation to OS and EFS

To evaluate the associations of post-treatment Node-RADS scores and clinical baseline characteristics with patient prognosis, univariable and multivariable Cox regression analyses were performed using OS and EFS as the endpoints, respectively (Table 3 and Table 4).

For OS, univariable Cox regression analysis showed that the neoadjuvant therapy regimen had borderline statistical significance (*p* = 0.067), while post-treatment Node-RADS was significantly associated with OS (*p* < 0.001). Based on the results of the univariable analysis and the clinical importance of age, relevant variables were included in the multivariable Cox regression model. Multivariable analysis demonstrated that both neoadjuvant therapy regimen and post-treatment Node-RADS remained statistically significant, indicating that they were independently associated with OS. Age was included in the model as an important clinical covariate for adjustment, although it did not reach statistical significance.

For EFS, univariable Cox regression analysis showed that pathological type (*p* = 0.039) and post-treatment Node-RADS (*p* < 0.001) were associated with EFS. Based on the univariable analysis results and the clinical importance of age, relevant variables were entered into the multivariable Cox regression model. The multivariable analysis showed that only post-treatment Node-RADS remained significantly associated with EFS, whereas pathological type did not retain statistical significance. Age was again included in the model as an adjustment covariate, but no significant association was observed.

These findings indicate that the post-treatment Node-RADS score was significantly associated with both OS and EFS and may serve as an important prognostic factor. The nomograms are shown in Supplementary Figures S2 and S3.

### 3.4. Comparison of Prognostic Model Performance and Kaplan–Meier Survival Analysis

**Figure 5:**
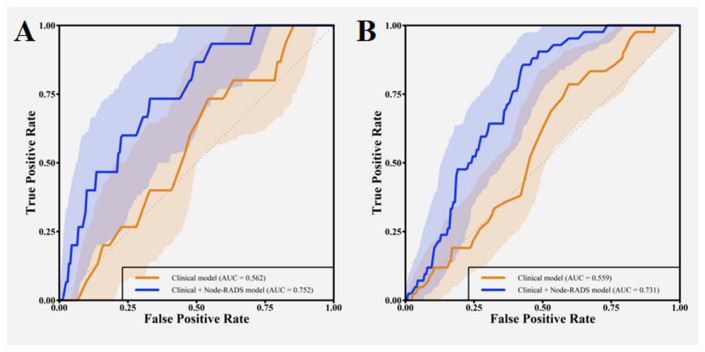
Prognostic performance of Node-RADS assessed by ROC analysis based on survival/event status at the last follow-up. (**A**) ROC curves for OS models with and without Node-RADS. (**B**) ROC curves for EFS models with and without Node-RADS.

To facilitate clinical risk stratification and comparison of survival curves, patients were divided into a high-risk group (Node-RADS score ≥ 3) and a low-risk group (Node-RADS score < 3) according to the risk stratification characteristics of the Node-RADS scoring system. In addition, to further evaluate the contribution of post-treatment Node-RADS to the discriminatory performance of prognostic models, we compared the predictive performance of the clinical model and the clinical plus post-treatment Node-RADS model for OS and EFS. Based on survival/event status at the last follow-up, the results showed that, after incorporation of post-treatment Node-RADS, the AUC of the OS prediction model increased from 0.562 to 0.752, and the AUC of the EFS prediction model increased from 0.559 to 0.731, indicating improved model discrimination (Figure 5 and Table 5).

**Figure 6:**
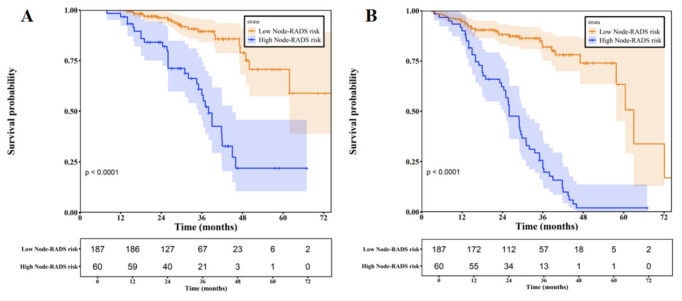
Kaplan–Meier survival curves stratified by Node-RADS risk group. (**A**) Overall survival. (**B**) Event-free survival.

The median follow-up duration was 29.7 months (interquartile range, 21.1–39.5 months). Kaplan–Meier survival analysis showed that patients in the high Node-RADS risk group had significantly worse OS and EFS than those in the low Node-RADS risk group. The median OS in the high Node-RADS risk group was 38.0 months (interquartile range [IQR], 26.0–46.0 months) whereas the median OS in the low Node-RADS risk group was not reached during follow-up. For EFS, the median EFS was 63.0 months (IQR, 47.1–72.1 months) in the low Node-RADS risk group and 26.0 months (IQR, 16.0–36.0 months) in the high Node-RADS risk group. The Kaplan–Meier survival curves for OS and EFS are shown in Figure 6.

## 4. Discussion

Although neoadjuvant therapy has markedly changed the perioperative management of resectable and locally advanced NSCLC, post-treatment lymph node status remains one of the key factors affecting surgical decision-making, recurrence risk, and long-term survival [13]. In this study, we focused on this clinical issue and systematically evaluated the predictive value of post-treatment Node-RADS combined with clinical baseline variables for lymph node metastasis and prognosis in patients with NSCLC after neoadjuvant therapy. The results showed that post-treatment Node-RADS was significantly associated with postoperative pathological lymph node metastasis, and the discriminatory performance of the lymph node metastasis prediction model was markedly improved after inclusion of this parameter. At the same time, post-treatment Node-RADS was also significantly associated with both OS and EFS, and it remained independently associated with prognosis in multivariable Cox regression analysis. These findings suggest that post-treatment Node-RADS not only reflects the residual lymph node tumor burden after neoadjuvant therapy, but also provides additional information for long-term risk stratification.

Accurate assessment of lymph node involvement is of critical importance. Conventional CT-based evaluation of lymph nodes has limited accuracy because benign lymph nodes may undergo reactive enlargement, whereas malignant lymph nodes may remain normal in size, thereby increasing diagnostic difficulty [14]. By integrating morphologic features and size characteristics of lymph nodes, the Node-RADS system provides a standardized risk score to reduce such uncertainty [7]. Its clinical value is not limited to a single tumor type. In bladder cancer, Leonardo et al. showed that Node-RADS was an independent predictor of lymph node invasion and demonstrated moderate-to-high overall accuracy. In addition, different cutoff values could be adapted to different clinical scenarios, suggesting that Node-RADS may be useful not only for diagnosis but also for risk stratification and decision support [15]. In cervical cancer, Ninkova et al. reported that Node-RADS provided standardized lymph node assessment, improved the diagnostic performance for predicting pathologic nodal (pN) status, and showed good usability and interobserver agreement [16]. In endometrial cancer, Bonatti et al. further confirmed that Node-RADS had good reproducibility and accuracy for regional lymph node staging, although its performance was influenced by reader experience, and MRI showed higher sensitivity than CT for lymph node assessment [17]. Taken together, these studies indicate that the core value of Node-RADS lies not merely in identifying enlarged lymph nodes, but in providing a transferable and standardized framework for assessing the risk of nodal malignancy across different solid tumors. In the field of lung cancer, the application of Node-RADS also has a practical basis. Meyer et al. found in a study of mediastinal lymph nodes in lung cancer that Node-RADS scores were significantly associated with the malignant status of mediastinal lymph nodes and could be used to distinguish benign from malignant lymph nodes [18]. However, compared with the general preoperative staging setting, reassessment of lymph nodes in NSCLC after neoadjuvant therapy is more complex, because treatment-related fibrosis, necrosis, inflammatory responses, and immune cell infiltration may all weaken the discriminatory ability of size-based criteria alone. Against this background, the present study further extended the application of Node-RADS from general lymph node stratification in lung cancer to the identification of residual nodal metastasis and the evaluation of long-term prognosis after neoadjuvant therapy in NSCLC. In this study, Node-RADS was used for patient-level risk stratification rather than node-by-node or station-by-station diagnostic matching; therefore, the final score should be interpreted as an imaging indicator of overall nodal risk rather than as proof of metastasis in a specific lymph node station. Accordingly, the diagnostic analysis in this study should be interpreted as a patient-level prediction of overall pathological nodal positivity, rather than as a node-specific or station-specific validation of Node-RADS. Nevertheless, the moderate sensitivity of the Clinical + Node-RADS model should be interpreted cautiously. Although the model showed acceptable specificity, its sensitivity for predicting residual lymph node metastasis remained limited, indicating that a low-risk Node-RADS result cannot reliably exclude pathological nodal involvement after neoadjuvant therapy. Therefore, post-treatment Node-RADS should not be used as a stand-alone criterion to omit systematic lymph node dissection or de-escalate surgical management. Instead, it should be regarded as an adjunctive imaging tool for perioperative risk stratification and multidisciplinary decision-making.

A deeper clinical value of the Node-RADS system lies in its potential to convey prognostic information. In lung cancer, existing evidence has mainly focused on the identification of benign and malignant mediastinal lymph nodes, but these studies also suggest that Node-RADS may be extended to prognostic assessment. Building on the work of Meyer et al. [18], the present study further expanded its application to preoperative reassessment and long-term risk stratification in NSCLC after neoadjuvant therapy. Our results showed that post-treatment Node-RADS was not only independently associated with OS and EFS, but also significantly improved model performance after incorporation, with the AUC for OS prediction increasing from 0.562 to 0.752 and the AUC for EFS prediction increasing from 0.559 to 0.731. Its prognostic value has also been supported in other tumor types. In a neoadjuvant treatment cohort of locally advanced gastric cancer, Sun et al. found that, after incorporation of Node-RADS into the integrated model, the net reclassification improvement (NRI) for 5-year OS and disease-free survival (DFS) was 0.379 and 0.364, respectively, while the integrated discrimination improvement (IDI) was 0.103 and 0.107, respectively. These findings suggest that Node-RADS not only provides staging information, but also a quantifiable gain in survival prediction [19]. Similarly, Okan Dilek et al. reported that Node-RADS showed good performance in predicting lymph node metastasis in patients with locally advanced gastric cancer after neoadjuvant therapy and also demonstrated certain prognostic value [20]. In papillary renal cell carcinoma, Li Xiaoxia et al. found that Node-RADS was an independent predictor of progression-free survival and cancer-specific survival [21]. In addition, Wang Yanjun et al. [22] developed a hyper-enhanced Node-RADS (HE-Node-RADS) model [23,24,25] in papillary thyroid carcinoma and evaluated the diagnostic performance of both Node-RADS and the modified model at different thresholds, showing that Node-RADS had moderate to good diagnostic efficacy. Taken together, previous studies suggest that Node-RADS has not only diagnostic value but also potential utility in prognostic assessment, and that it has already shown promising applicability in the neoadjuvant treatment setting. Accordingly, the significance of Node-RADS in the present study extends beyond simple radiologic stratification of the probability of malignancy. Rather, it may serve as an adjunctive imaging indicator reflecting residual nodal risk and the possibility of long-term adverse outcomes after therapy.

This study has several limitations. First, although the analysis was based on data from three centers, the retrospective design still carries an inherent risk of potential bias in patient selection and data collection. Second, the sample size was relatively limited, and the study population may still represent a relatively homogeneous cohort; therefore, the generalizability and robustness of the proposed diagnostic and prognostic models require further validation in larger, independent, and more diverse populations. Third, the number of patients receiving chemotherapy alone was small compared with those receiving chemoimmunotherapy, which may have introduced potential bias into the survival analyses and limited the reliability of subgroup analyses according to neoadjuvant treatment regimen. Although parsimonious adjusted models were used, potential overfitting could not be completely excluded because of the limited sample size and number of outcome events. Fourth, this study included both clinically node-negative and clinically node-positive patients before neoadjuvant therapy; therefore, the diagnostic performance of post-treatment Node-RADS should be interpreted in the context of a heterogeneous real-world neoadjuvant surgical cohort. Fifth, ROC analyses for OS and EFS were based on survival/event status at the last follow-up, and future studies using fixed-time time-dependent ROC analyses may provide a more refined evaluation of prognostic performance. Sixth, this study did not perform a head-to-head comparison between post-treatment Node-RADS and other approaches such as radiomics; therefore, its incremental value and generalizability still require further investigation. Finally, the present analysis was based on an overall patient-level score and therefore could not fully reflect the spatial heterogeneity across different lymph node stations. Further validation using node-by-node or station-by-station pathological matching is warranted.

In conclusion, the Node-RADS system may provide useful imaging information for patient-level lymph node metastasis assessment and prognostic stratification in patients with NSCLC after neoadjuvant therapy, although further validation using node-by-node or station-by-station pathological matching is warranted. Its association with post-treatment survival outcomes suggests that Node-RADS may serve as an adjunctive imaging indicator for perioperative individualized risk stratification and treatment decision-making.

## Figures and Tables

**Table 1:** Baseline clinical characteristics of the patients.

Variables	Total (*n* = 247)
**Gender, ** ***n*** ** (%)**	
Female	36 (14.6)
Male	211 (85.4)
**Age (Mean ± SD)**	63.40 ± 7.58
**Hypertension, ** ***n*** ** (%)**	
No	172 (69.6)
Yes	75 (30.4)
**Diabetes, ** ***n*** ** (%)**	
No	208 (84.2)
Yes	39 (15.8)
**Coronary heart disease, ** ***n*** ** (%)**	
No	224 (90.7)
Yes	23 (9.3)
**Cerebral infarction, ** ***n*** ** (%)**	
No	231 (93.5)
Yes	16 (6.5)
**Clinical stage, ** ***n*** ** (%)**	
IB	25 (10.1)
IIA	23 (9.3)
IIB	40 (16.2)
IIIA	103 (41.7)
IIIB	56 (22.7)
**Pathological type, ** ***n*** ** (%)**	
Squamous cell carcinoma	192 (77.7)
Adenocarcinoma	55 (22.3)
**Surgical method, ** ***n*** ** (%)**	
Open chest	118 (47.8)
VATS	129 (52.2)
**Blood loss (Mean ± SD)**	167.87 ± 287.65
**Number of neoadjuvant treatment cycles (Mean ± SD)**	2.64 ± 1.15
**Neoadjuvant therapy regimen, ** ***n*** ** (%)**	
Chemotherapy	18 (7.3)
Chemoimmunotherapy	229 (92.7)
**Chemotherapy regimen, ** ***n*** ** (%)**	
Paclitaxel combined with platinum	200 (81.0)
Pemetrexed combined with platinum	42 (17.0)
Gemcitabine combined with platinum	5 (2.0)
**Complications of neoadjuvant therapy, ** ***n*** ** (%)**	
No	85 (34.4)
Yes	162 (65.6)
**Post-treatment Node-RADS, ** ***n*** ** (%)**	
1	128 (51.8)
2	59 (23.9)
3	24 (9.7)
4	21 (8.5)
5	15 (6.1)

**Node-RADS**, Lymph Node Reporting and Data System; **SD**, standard deviation; **Mean**, Mean value.

**Table 2:** Predictive performance of Node-RADS for lymph node metastasis.

Model	AUC (95% CI)	Sensitivity (95% CI)	Specificity (95% CI)	Accuracy (95% CI)	PPV (95% CI)	NPV (95% CI)
**Clinical model**	0.570 (0.493–0.646)	0.013 (0.000–0.048)	0.994 (0.981–1.000)	0.684 (0.628–0.741)	0.500 (0.000–1.000)	0.686 (0.630–0.741)
**Clinical + Node-RADS model**	0.769 (0.704–0.834)	0.462 (0.353–0.576)	0.876 (0.828–0.927)	0.745 (0.700–0.798)	0.632 (0.508–0.776)	0.779 (0.728–0.837)

**Table 3:** Univariable Cox regression analyses of OS and EFS.

Variables	OS		EFS	
	HR (95% CI)	*p*	HR (95% CI)	*p*
**Gender**				
Female	1.00 (Reference)		1.00 (Reference)	
Male	1.15 (0.52–2.56)	0.731	1.12 (0.61–2.06)	0.718
**Age**	1.01 (0.98–1.05)	0.488	1.02 (0.99–1.05)	0.224
**Hypertension**				
No	1.00 (Reference)		1.00 (Reference)	
Yes	0.97 (0.53–1.77)	0.92	1.25 (0.80–1.94)	0.328
**Diabetes**				
No	1.00 (Reference)		1.00 (Reference)	
Yes	0.92 (0.43–1.98)	0.839	0.82 (0.46–1.48)	0.517
**Coronary heart disease**				
No	1.00 (Reference)		1.00 (Reference)	
Yes	0.57 (0.20–1.61)	0.292	0.58 (0.26–1.28)	0.176
**Cerebral infarction**				
No	1.00 (Reference)		1.00 (Reference)	
Yes	1.02 (0.32–3.30)	0.968	1.68 (0.84–3.37)	0.143
**Pathological type**				
Squamous cell carcinoma	1.00 (Reference)		1.00 (Reference)	
Adenocarcinoma	1.33 (0.71–2.51)	0.375	1.64 (1.03–2.61)	0.039
**Surgical method**				
Open chest	1.00 (Reference)		1.00 (Reference)	
VATS	0.63 (0.35–1.11)	0.108	1.06 (0.69–1.61)	0.796
**Blood loss**	1.00 (1.00–1.00)	0.421	1.00 (1.00–1.00)	0.583
**Number of neoadjuvant treatment cycles**	1.01 (0.75–1.36)	0.954	1.09 (0.92–1.31)	0.325
**Neoadjuvant therapy regimen**				
Chemotherapy	1.00 (Reference)		1.00 (Reference)	
Chemoimmunotherapy	3.25 (0.92–11.47)	0.067	1.39 (0.65–2.98)	0.393
**Chemotherapy regimen**				
Paclitaxel combined with platinum	1.00 (Reference)		1.00 (Reference)	
Pemetrexed combined with platinum	1.35 (0.69–2.63)	0.383	1.53 (0.93–2.53)	0.096
Gemcitabine combined with platinum	0.00	0.995	0.00	0.996
**Complications of neoadjuvant therapy**				
No	1.00 (Reference)		1.00 (Reference)	
Yes	1.21 (0.68–2.16)	0.522	1.24 (0.79–1.93)	0.350
**Post-treatment Node-RADS**	1.62 (1.36–1.92)	<0.001	1.40 (1.22–1.61)	<0.001

**Table 4:** Multivariable Cox regression analyses of OS and EFS.

Variables	OS		EFS	
	HR (95% CI)	*p*	HR (95% CI)	*p*
**Age**	1.03 (1.00–1.07)	0.090	1.02 (0.99–1.05)	0.155
**Pathological type**				
Squamous cell carcinoma			1.00 (Reference)	
Adenocarcinoma			1.57 (0.98–2.52)	0.0582
**Neoadjuvant therapy regimen**				
Chemotherapy	1.00 (Reference)			
Chemoimmunotherapy	7.50 (1.94–28.99)	0.004		
**Post-treatment Node-RADS**	1.79 (1.50–2.15)	<0.001	1.41 (1.23–1.62)	<0.001

**Table 5:** ROC curve analysis of OS and EFS based on survival/event status at the last follow-up.

Model	OS			EFS		
	AUC	95% CI	*p*	AUC	95% CI	*p*
**Clinical model**	0.562	0.426–0.698	0.0498	0.559	0.469–0.649	0.0013
**Clinical + Node-RADS model**	0.752	0.636–0.867	<0.001	0.731	0.657–0.805	<0.001

## Data Availability

The original contributions presented in this study are included in the article/Supplementary Materials. Further inquiries can be directed to the corresponding author.

## Supplementary Materials

The following supporting information can be downloaded at https://www.mdpi.com/article/10.3390/diagnostics16132021/s1, Figure S1. Nomogram for the prediction of lymph node metastasis in non-small cell lung cancer after neoadjuvant therapy. Figure S2. Nomogram for the prediction of OS in non-small cell lung cancer after neoadjuvant therapy. Figure S3. Nomogram for the prediction of EFS in non-small cell lung cancer after neoadjuvant therapy. Table S1 Candidate variables for multivariable logistic regression analysis of lymph node metastasis. Table S2. Patient-level frequency of pN+ disease and category-specific odds ratios according to Node-RADS category. Table S3. Univariable and multivariable logistic regression analyses of lymph node metastasis.
