# Supplementary material for: Prognostic and Diagnostic Value of Node-RADS for Non-Small Cell Lung Cancer Following Neoadjuvant Therapy: A Multicenter Cohort Study
Source: Diagnostics (Basel). 2026 Jun 29;16(13):2021. doi: 10.3390/diagnostics16132021 (PMC13360237; doi:10.3390/diagnostics16132021)
Supplement: Supplementary file 1 [file diagnostics-16-02021-s001.zip › Supplementary Figure.pdf]

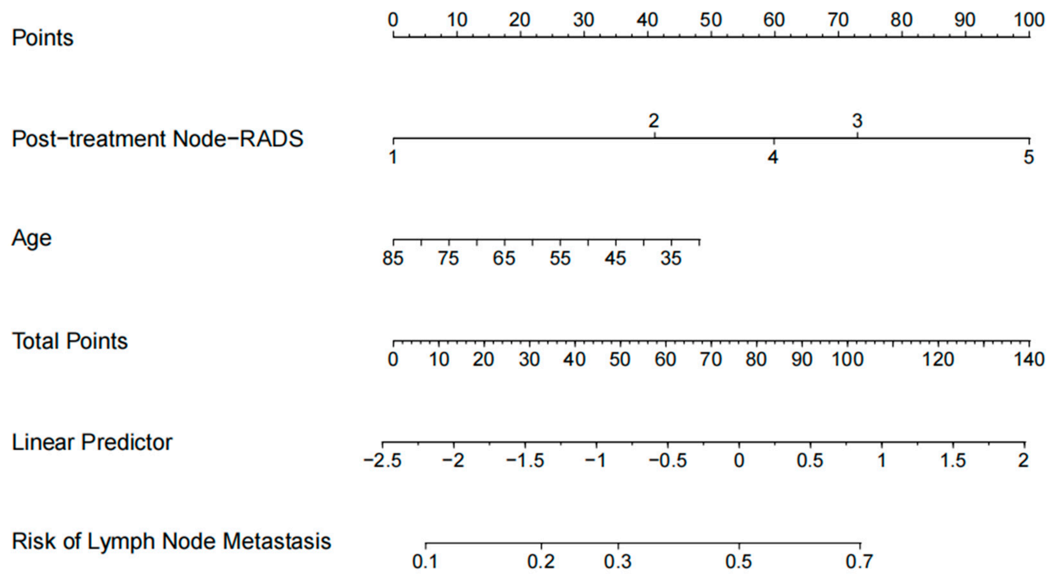

Supplementary Figure S1 Nomogram for the prediction of lymph node metastasis in non-small cell lung cancer after neoadjuvant therapy

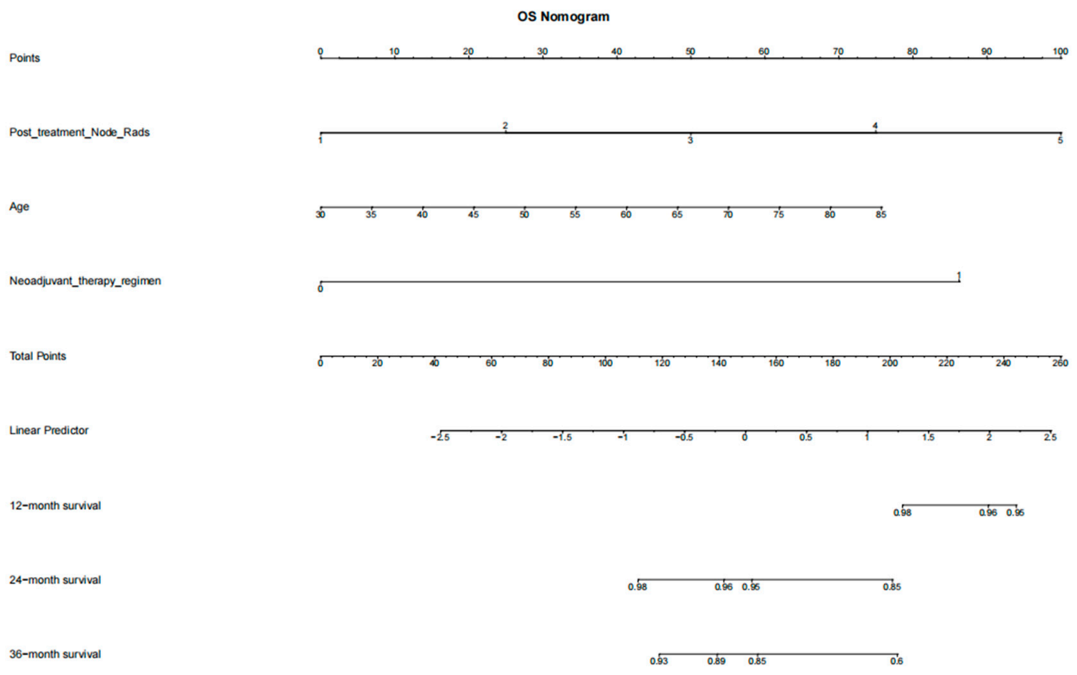

Supplementary Figure S2 Nomogram for the prediction of OS in non-small cell lung cancer after neoadjuvant therapy

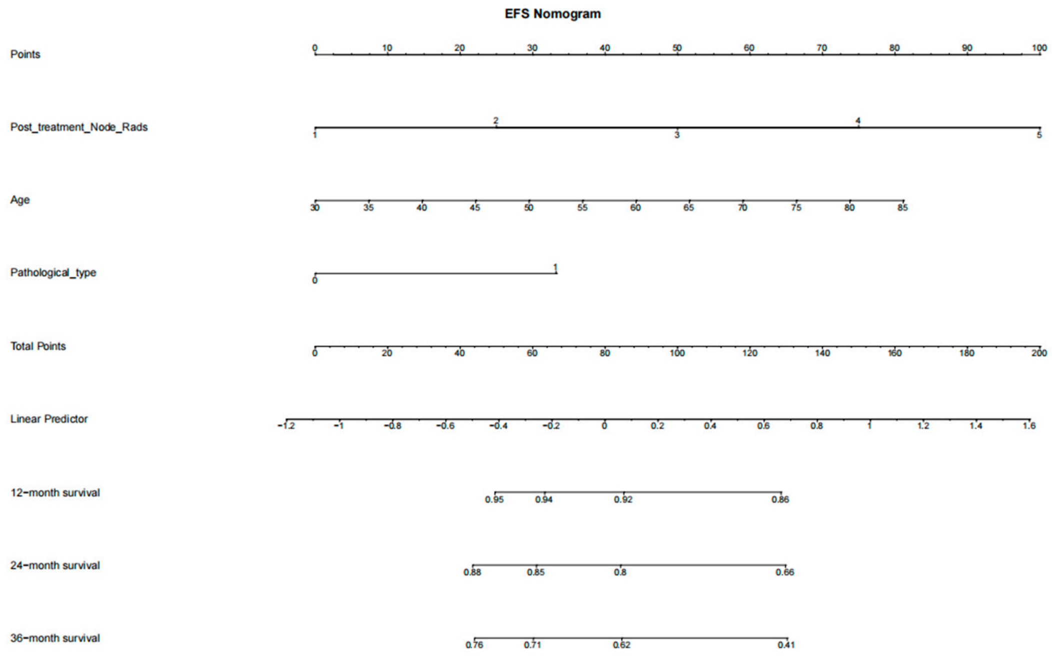

Supplementary Figure S3 Nomogram for the prediction of EFS in non-small cell lung cancer after neoadjuvant therapy
