# Supplementary material for: Prognostic and Diagnostic Value of Node-RADS for Non-Small Cell Lung Cancer Following Neoadjuvant Therapy: A Multicenter Cohort Study
Source: Diagnostics (Basel). 2026 Jun 29;16(13):2021. doi: 10.3390/diagnostics16132021 (PMC13360237; doi:10.3390/diagnostics16132021)
Supplement: Supplementary file 1 [file diagnostics-16-02021-s001.zip › Supplementary Table.pdf]

Supplementary Table S1 Candidate variables for multivariable logistic regression analysis of lymph node metastasis

Variables	Multivariate logistics regression	
	**OR (95% CI)**	**P**
**Age**	0.972(0.933 - 1.012)	0.167
**Pathological type**		
Squamous cell carcinoma	1.000(Reference)	
Adenocarcinoma	2.722(0.832 - 8.908)	0.098
**Number of neoadjuvant treatment cycles**	0.849(0.598 - 1.206)	0.361
**Neoadjuvant therapy regimen**		
Chemotherapy	1.000(Reference)	
Chemoimmunotherapy	0.978(0.270 - 3.540)	0.972
**Chemotherapy regimen**		
Paclitaxel combined with platinum	1.000(Reference)	
Pemetrexed combined with platinum	1.096(0.298 - 4.028)	0.890
Gemcitabine combined with platinum	3.707(0.419 - 32.769)	0.239
**Complications of neoadjuvant therapy**		
No	1.000(Reference)	
Yes	0.600(0.309 - 1.166)	0.132
**Post-treatment Node-RADS**		
1	1.000(Reference)	
2	4.145(1.922 - 8.939)	<0.001
3	12.127(4.332 - 33.944)	<0.001
4	6.449(2.243 - 18.535)	<0.001
5	21.202(5.192 - 86.583)	<0.001

Supplementary Table S2. Patient-level frequency of pN+ disease and category-specific odds ratios according to Node-RADS category

Node-RADS category	Total patients, n(%)	pN+ patients,n(%)	OR (95% CI)
1	128 (51.8)	1 (0.8)	Reference
2	59 (23.9)	17 (28.8)	35.00 (6.37-192.21)
3	24 (9.7)	24 (100.0)	4165.00 (164.80-105262.01)
4	21 (8.5)	21 (100.0)	3655.00 (144.13-92686.03)
5	15 (6.1)	15 (100.0)	2635.00 (102.81-67537.55)

Supplementary Table S3 Univariable and multivariable logistic regression analyses of lymph node metastasis

**Variables**	**Lymph Node Metastasis**			
	**Univariate logistics regression**		**Multivariate logistics regression**	
	**OR (95% CI)**	**P**	**OR (95% CI)**	**P**
**Gender**				
Famale	1.000(Reference)			
Male	0.684(0.329-1.422)	0.309		
**Age**	0.972(0.939-1.007)	0.120	0.973(0.935-1.011)	0.165
**Hypertension**				
No	1.000(Reference)			
Yes	1.123(0.629-2.003)	0.695		
**Diabetes**				
No	1.000(Reference)			
Yes	1.100(0.531-2.277)	0.797		
**Coronary heart disease**				
No	1.000(Reference)			
Yes	0.427(0.140-1.300)	0.134		
**Cerebral infarction**				
No	1.000(Reference)			
Yes	1.325(0.464-3.785)	0.599		
**Pathological type**				
Squamous cell carcinoma	1.000(Reference)			
Adenocarcinoma	2.186(1.178-4.054)	0.013		
**Surgical method**				
Open chest	1.000(Reference)			
VAST	0.946(0.553-1.619)	0.840		
**Blood loss**	1.000(0.999-1.001)	0.796		
**Number of neoadjuvant treatment cycles**	0.784(0.582-1.056)	0.109		
**Neoadjuvant therapy regimen**				
Chemotherapy	1.000(Reference)			
Chemoimmunotherapy	0.338(0.128-0.893)	0.029		
**Chemotherapy regimen**				
Paclitaxel combined with platinum	1.000(Reference)			
Pemetrexed combined with platinum	2.338(1.185-4.613)	0.014		
Gemcitabine combined with platinum	1.714(0.279-10.534)	0.561		
**Complications of neoadjuvant therapy**				

No	1.000(Reference)			
Yes	0.559(0.321-0.974)	0.040		
**Post-treatment Node-RADS**				
1	1.000(Reference)		1.000(Reference)	
2	3.634(1.758-7.509)	<0.001	3.701(1.783-7.681)	<0.001
3	10.185(3.880-26.735)	<0.001	10.209(3.868-26.946)	<0.001
4	6.722(2.496-18.106)	<0.001	6.724(2.487-18.182)	<0.001
5	24.444(6.275-95.220)	<0.001	24.123(6.165-94.388)	<0.001
